# Plasma Charge Current for Controlling and Monitoring Electron Beam Welding with Beam Oscillation

**DOI:** 10.3390/s121217433

**Published:** 2012-12-14

**Authors:** Dmitriy Trushnikov, Vladimir Belenkiy, Valeriy Shchavlev, Anatoliy Piskunov, Aleksandr Abdullin, Georgy Mladenov

**Affiliations:** 1 Perm National Research Polytechnic University, 29 Komsomolsky Av., Perm 614990, Russian Federation; E-Mails: vladimirbelenkij@yandex.ru (V.B.); sovokg@gmail.com (V.S.); pal2010@yandex.ru (A.P.); zuac@perm.ru (A.A.); 2 Institute of Electronics, Bulgarian Academy of Sciences, 72 Tzarigradsko Chaussee, Sofia 1784, Bulgaria; E-Mail: gmmladenov@abv.bg

**Keywords:** electron beam welding, electron beam oscillation, plasma charge current, beam focus control, keyhole

## Abstract

Electron beam welding (EBW) shows certain problems with the control of focus regime. The electron beam focus can be controlled in electron-beam welding based on the parameters of a secondary signal. In this case, the parameters like secondary emissions and focus coil current have extreme relationships. There are two values of focus coil current which provide equal value signal parameters. Therefore, adaptive systems of electron beam focus control use low-frequency scanning of focus, which substantially limits the operation speed of these systems and has a negative effect on weld joint quality. The purpose of this study is to develop a method for operational control of the electron beam focus during welding in the deep penetration mode. The method uses the plasma charge current signal as an additional informational parameter. This parameter allows identification of the electron beam focus regime in electron-beam welding without application of additional low-frequency scanning of focus. It can be used for working out operational electron beam control methods focusing exactly on the welding. In addition, use of this parameter allows one to observe the shape of the keyhole during the welding process.

## 1. Introduction

Electron beam welding (EBW) is a fusion welding process often done in a vacuum. The process has a number of advantages: high power concentration in the electron beam, easy control of the energy flow into the metal, smaller heat-affected areas, equal strength of the weld joint and main metal, *etc.* These advantages allow the use of electron beams for welding reactive and nonferrous metals, high-tensile and heat-resistant alloys that are typically used in the production of critical products.

However, certain problems arise in the EBW process, which are related to instability of weld-joint formation and difficulties in creating and controlling an optimal focus regime. A major limitation in controlling such a focus regime is the lack of understanding of the processes occurring during EBW. The complex character and high speed of these processes make numerical modeling very difficult, forcing researchers to rely on experimental research methods.

The basic parameters of EBW are accelerating voltage, electron beam current, focusing-coil current, welding speed, operating distance, vacuum level in the process chamber, *etc.* These parameters are chosen according to diverse factors such as the operator’s own experience, mathematical models [1,2], or expensive and hard statistical analysis [3–5]. The most difficult parameter to identify and reproduce in EBW is the focusing mode. The operator of an EBW set needs to manually focus the beam and adjust the focusing-coil current based on the luminosity brightness from the operational area of the beam aimed at the target material, e.g., wolfram (tungsten). When the luminosity brightness becomes maximal, the focusing mode is considered sharp [6].

The manual interpretation process is subjective and prone to errors. Each operator interprets the luminosity brightness of the operational area differently and, therefore, the results of each weld are hard to reproduce. Changing the focusing current by only 1% may cause a 20–60% fluctuation of fusion depth. The focusing mode also significantly influences the probability of various defects specific to EBW such as spiking, cavitation, medial cracks, *etc.* The difficulties in focusing control are aggravated by changes in the electronic and optical systems of an electronic beam projector due to cathode wear and tear or after planned maintenance.

In recent years, this problem has been partly solved by using a modified Faraday cup to control the electronic beam density distribution [7,8]. During circular scanning, the beam passes through a set of radial gaps in the wolfram disk. After the current passing through the gaps is measured, the density of the electronic-beam power, beam diameter, maximum specific power and other important metrics are calculated based on computer tomography algorithms. By controlling these basic parameters of the electronic beam, the parameters of the welding seams can be reproduced. It has also been reported that this technology can be migrated between various electron beam sets [9]. Some vacuum chambers, however, do not support internal mounting of the required sensor (the Faraday cup). Also, to avoid sensor corruption, this method is often used for controlling the beam focus at low power before the welding process starts. For welding operation modes, the focusing current should be adjusted based on experiments with various materials, thicknesses and types of electronic-beam projectors. Moreover, the systems based on this method do not support real-time control and adjustment of the focusing mode during welding. Real-time adjustments are important for welding large objects especially when the cathode capabilities of electron emission adjustments and thus electronic and optic adjustments needed, are significant.

Therefore, the control, monitoring and analysis of the processes accompanying EBW requires analysis of some secondary signal parameters, such as secondary electron or ion emission, optical emission, X-rays, *etc.*

Popular focus-control methods include systems for measuring the secondary emission parameters while scanning the joint being welded [10–12]. Sharp focusing is determined based on the maximum amplitude of the secondary signal’s peaks when the beam crosses the joint. Similar to this system are the raster scanning systems that register the signal of reflected electrons [12]. These methods allow the electronic beam focus to be preset at low power before welding starts. However, for operating welding modes, the focusing current should be adjusted experimentally depending on the materials, thickness and types of electron beam guns used.

Other control methods are based on the correlation between secondary emission parameters in the welding area and the specific power of the electron beam. In [13], X-rays are used to control the EBW parameters. In [14,15], there is a description of the correlation between focusing mode and the average values and amplitudes of the reflected and secondary currents, ionic current, as well as X-ray density. These results helped Mitsubishi Electric Corporation develop automatic electronic-beam focus control systems [15], which use electronic-beam with focusing control at low power as reference points.

One of the specific processes caused by the impact of the dense electron beam to the metal during EBW is the formation of plasma in the operational area [16–21]. The parameters of the plasma are closely connected with the electron beam thermal effect on the metal being welded. In [22–27], plasma current parameters are suggested for electron beam focusing control.

All the above methods use extreme correlations between the secondary emissions and the focusing coil current. These correlations are characterized by dead zones and two values of the focusing coil current that ensure similar signal parameters. That is why the adaptive electronic-beam focus stabilization systems support low-frequency scanning [28], which significantly limits their performance and welding joint quality.

In recent years, control and monitoring of electron beam and laser-based welding has become more and more popular [29–38]. Laser technologies and electron beam welding are based on similar principles. New research opportunity provided by modern signal processing is finding an increased interest by researchers. Older methods only allowed inspection of amplitude ratios, while today we can analyze the structure of the secondary current signal in the plasma during EBW [39,40]. It has been reported that the signal consists of a short high-frequency random series of signals passing one by one. However, the impulses of the currents are significant (up to 0.5 A).

One of the well-known ways to increase the signal/noise ratio for physical object analysis is periodic exposure of the object with subsequent analysis of its response at a given exposure frequency [40]. In [25,38,39], it is reported that periodic exposure of the electron beam (beam current modulation or oscillation) causes normalization of the waveform in the fusion channel created by the electron beam, thus making the series of impulses passing at constant intervals proportional to the waveform frequency. However, this research is not deemed sufficiently reliable, so further investigation is required. For example, simultaneous signal recording in the deflector coils and of the secondary current signals in the plasma was not performed, while the oscillation trajectory used was too complicated for reliable analysis. Also, the principles for generating harmonics proportional to the oscillation frequency were not revealed.

This article studies the behavior of the current in the plasma formed in the operational area of the electron beam, when using EBW and beam oscillation, based on the coherent accumulation method (cross-correlation analysis) [40,41]. This method can be used to obtain not only amplitude ratios, but also the phase ones, as well as determining how the current signals in the plasma are synchronized with the deflecting coil signals during EBW. These results can be useful for developing oscillation-type selection methods and methods to control EBW against the parameters of the plasma current.

## 2. Experimental Section

### 2.1. Experimental Set Up

In order to get generalized results, beam sweeps of the simplest trajectories, along and across the joint, were used. The signal from the deflecting coil current had a linear shape. The following four factors were varied: (i) electron beam oscillation amplitude, (ii) oscillation frequency, (iii) welding power, and (iv) focus current. Samples of chrome-molybdenum steel with 0.15% carbon, 5% chrome, and around 1% molybdenum were welded at an accelerating voltage of 60 kW and a welding speed of 5 mms^−1^. In the case of adding oscillations to the stationary beam position, we add to the basic process parameters the beam current, the energy of the beam electrons, the welding speed, the focusing current and the distance from the electron gun to the work-piece surface. Three new parameters are studied: amplitude, frequency and the kind of trajectory. In planned study, there are a lot of variables, demanding many experiments; nevertheless that one can use optimal design of experiments to minimize this number. Factor variations for each series is specified in Table 1. The table uses the following notations: *P* – welding power, *ΔI_f_* = *I_f_* – *I_f_*_0_ – focus degree (difference between current focus values in welding with beam oscillation and sharp-focus current, which provides maximal welding depth without beam oscillation), 2*A* – duplicated sweep amplitude, *f* – oscillation frequency. Total number of experiments performed in different modes of welded samples was 83.

Transverse metallurgical sections of the weld were made from all the welded samples. The focus regime was determined by the transverse sizes of the penetration depth. The sharp focus regime helps in maximum penetration depth. The weld penetration depth with sharp focusing and without electron-beam oscillation was equal to 10...16 mm.

**Figure 1.**
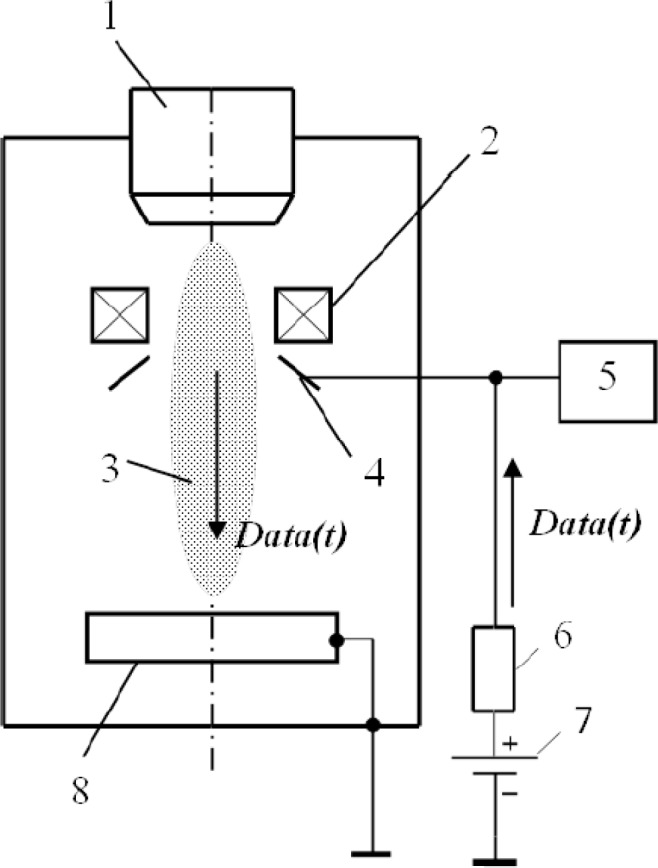
Scheme of plasma charge current registration: 1—electron gun, 2—focusing lens, 3—plasma formed over the area of electron beam welding, 4—electron collector, 5—a system of registration, 6—load resistor, 7—a source of bias, 8–—work piece, *Data(t)*—secondary current signal.

Over a welding zone, the collector in the form of a disc with an orifice is situated at the outlet of the electron beam gun in the coaxial direction in relation to the beam conductor (Figure 1). The collector had a positive potential of 50 V. Resistance of loading was 50 Ω.

In making welding passes, a computer information measuring system based on an IBM compatible computer, fitted with a multichannel analogue-digital interface, is used for recording the current passing through the circuit of the collector. The recorded results are stored in a file for further processing. During experiments, the range of sampling frequency ranged from 100 kHz to 1 MHz per channel.

### 2.2. Coherent Accumulation Method

Time sequence research often reveals the mutual influence of processes in conditions when the signals are shifted by a certain time period (the*τ* lag), the influence occurs with some delay or outrun. To detect this time lag, we should analyze the signals and determine their distinctive characteristics. In some cases, the time lag is obvious, e.g., when we switch on an electric heater, its temperature rises with a delay that can be calculated based on transient thermal analysis. When we switch off the heater, its temperature falls with some delay. However, in most cases we can determine neither the exact value of the time lag nor its presence.

The relation between any two random processes within a timeframe is characterized by their consistent correlation function (which is also called the cross-correlation function) [41,42]:

(1) $$r_{xy}\left(\tau \right)=\underset{t_{0}\to \infty }{\text{lim}}\int_{0}^{t_{0}}y\left(t\right)\cdot x\left(t+\tau \right)dt$$

where *t*_0_ is a sample time.

For periodic signals, cross-correlation analysis is used only when their periods are aliquot. In this case, the *r_xy_(τ)* function will also be periodic, and the number of harmonics will characterize the ratio of the periods of the original functions.

To analyze the secondary signal, we used the coherent accumulation method, which is a special case of cross-correlation analysis [41]. This method is widely used for directing an electron beam towards a joint [43], but it has not been applied before for focus control.

For implementation of the coherent accumulation method, we generated a square basis signal of period *g(t)* with a low relative duration based on the periodic signal of the deflection coil with the same period *T*. Mathematically speaking, we can describe *g(t)* generation as follows. First, the constant component *Osc*1(*t*) = *Osc*(*t*) – 〈*Osc*(*t*)〉 (where 〈*Osc*(*t*)〉 is an average of the *Osc*(*t*) signal), was filtered from the deflection coil signal *Ocs*(*t*). Next, we defined an intermediary function *Osc*2(*t*) = *Osc*1(*t –* Δ*T*) shifted from *Osc1*(*t*) by a percentage of the period Δ*T*. Δ*T* will then be used for calculating the impulse width of the basis signal *g(t)*.

Then, we defined the basis function as:

(2) $$G\left(t\right)=\{\begin{array}{ll} 1, & \left\{\left(\mathrm{Osc}1\left(t-\Delta T/2\right)\geq 0\right)\cap \left(\mathrm{Osc}2\left(t-\Delta T/2\right)\leq 0\right)\right\}\\ 0, & \text{otherwise}. \end{array}$$

**Figure 2.**
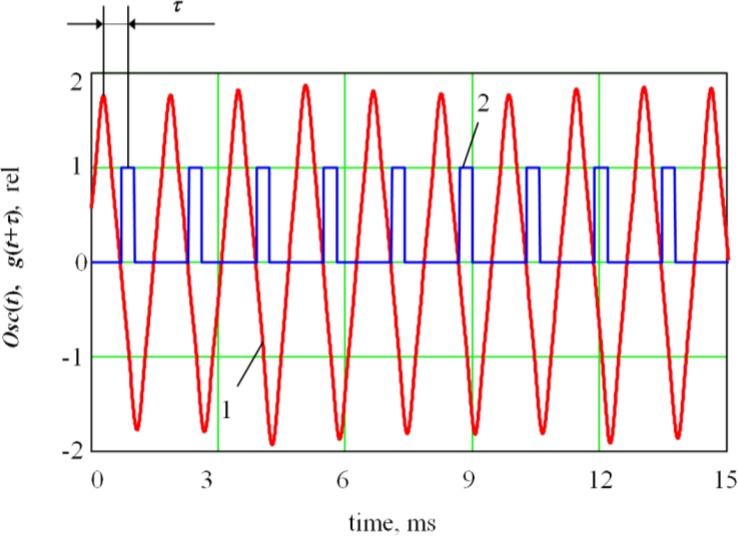
Formation of the basis signal *g*(*t+τ*): ***1****a*s a waveform of the deflection coil current *Osc*(*t*); ***2*** is a formed basis signal *g*(*t+τ*).

The resulting function *G(t)* is a set of impulses for the points where the *Osc*1(*t*) function is zero, so it can be used as a basis function. However, for better visibility, we associated the impulses with the maximum values of the deflection coil signals. The *G(t)* function was shifted by one-fourth of its period: *g*(*t*) = *G*(*t* – *T*/4). The resulting basis signal *g(t)* shifted by *τ* from the deflection coil signal *Osc*(*t*) is shown in (Figure 2).

Then, we calculated the coherent accumulation value by multiplying the basis signal *g*(*t+τ*) shifted by *τ* by the secondary current signal in the plasma *Data*(*t*), and we performed time integration:

(3) $$S\left(\tau \right)=\frac{\int_{0}^{t_{0}}g\left(t-\tau \right)\cdot \mathrm{Data}\left(t\right)dt}{\frac{t_{0}}{T}\cdot \Delta T}$$

where *t*_0_ is a sample time (*t*_0_ >> *T*); *τ* (*0 < τ < T*, where *T* is a sweep period). The denominator equals Δ*T· t*_0_ /*T*, as this value is a measure of the support of the distribution of signal *Data*(*t*) [44].

**Figure 3.**
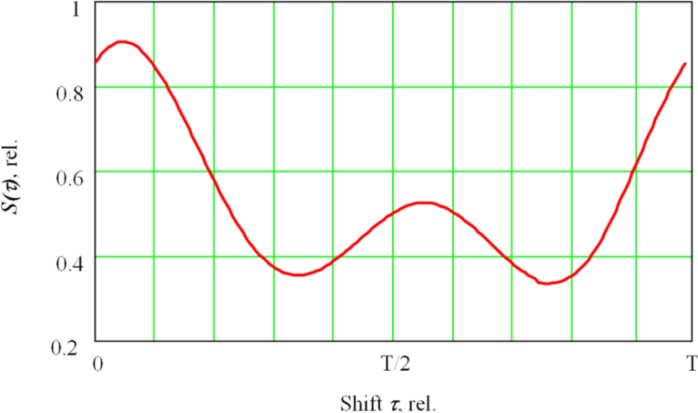
Function *S(τ)* which describes coherent accumulation result based on the reference input shift *τ*. Here, T is a sweep period.

The resulting function *S*(*τ*) (Figure 3) characterizes the average of the secondary signal for each *τ* shift, thus helping to analyze how the secondary current signal in the plasma is synchronized with the deflection coil signals *Osc*(*t*), or (which is the same)—with the deflection value of the oscillating electron beam in the fusion channel.

## 3. Results and Discussion

**Figure 4.**
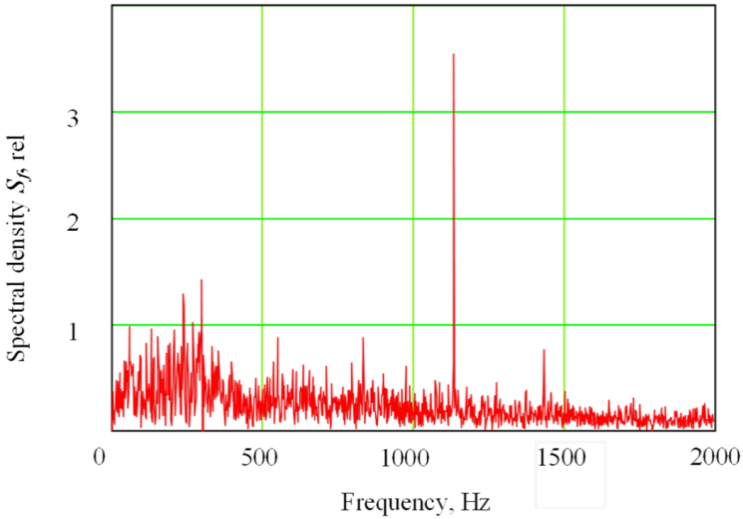
Low-frequency spectrum area of secondary current in plasma at the electron beam welding with electron beam oscillation across the joint (electron beam power *P* = 2.5 kW, sharp focus (Δ*I_f_* = 0), oscillation frequency *f* = 561 Hz, sweep size 2*A* = 0.9 mm).

Figure 4 shows a low-frequency spectral region of the secondary current signal in plasma electron with electron-beam oscillation across the joint. A peak is clearly visible at the frequency equal to the duplicated oscillation frequency. In particular, the second harmonic in the signal always peaks at EBW with oscillation across the joint. Both second and first harmonics were clearly observed in the secondary-emission signal at the welding process with the oscillation along the joint, while their ratio depended on the focus mode. The spectrum and waveforms of the secondary current in plasma during EBW are more fully described elsewhere in literature [24–27,39,40]. In the following study, research into the secondary signal was conducted using coherent accumulation.

**Figure 5.**
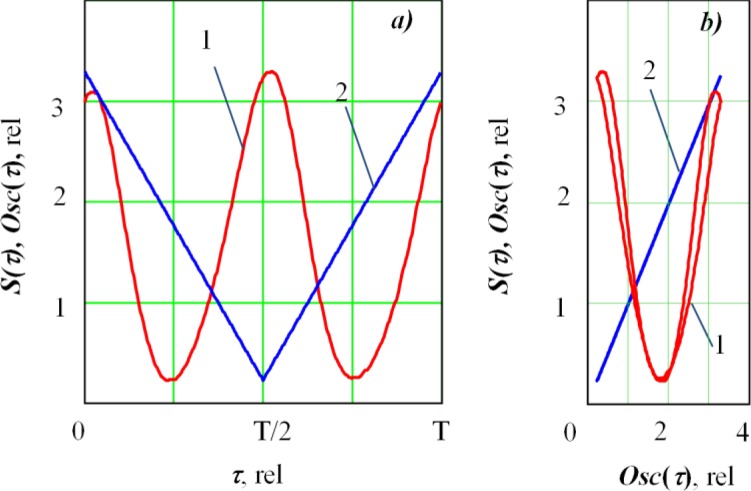
(**a**) Function *S(τ)* resulted from secondary signal processing with the coherent accumulation method on *τ*, and (**b**) *S(τ)* in phase space. 1—Function *S(τ)*, 2—is deflection coils current signal *Osc(t)*. It is the welding with oscillation across the joint (*P* = 2.5 kW, sharp focus (Δ*I_f_* = 0), oscillation frequency *f* = 563 Hz, sweep size 2*A* = 0.9 mm).

Figure 5(a) shows the result of processing the secondary emission current in the plasma signal by the accumulation method. This function can be presented in phase space (Figure 5(b)). For this purpose, we postponed a current of the deflection coils Osc(*τ)* or displacement of the electron beam in the keyhole on a horizontal axis. The resulting function *S*(*τ*) is practically symmetrical. When the beam is shifted on the side of the keyhole, there is an increase in the interaction area between the electron beam and the metal, leading to an increase in the secondary signal.

**Figure 6.**
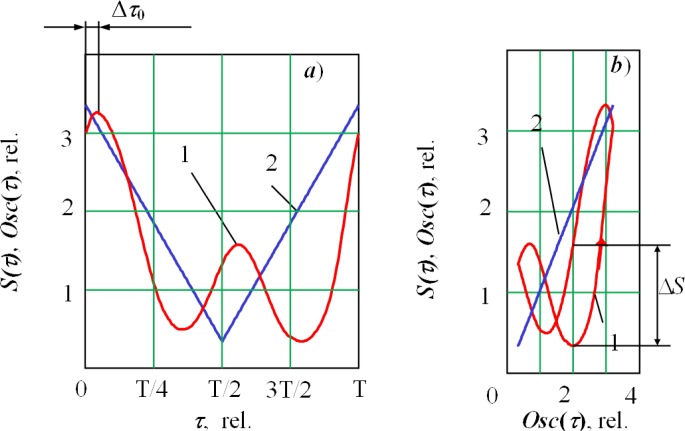
(**a**) Function *S(τ)* resulted from secondary signal processing with the coherent accumulation method on *τ* and (**b**) *S(τ)* in phase space. 1—Function *S(τ)*, 2—is deflection coils current signal *Osc(t)*. It is the welding with oscillation across the joint (*P* = 2.5 kW, underfocused regime (Δ*I_f_* = −15 mA), oscillation frequency *f* = 645 Hz, sweep size 2*A* = 1.5 mm).

Figure 6 presents the results for oscillation along the joint. The asymmetry of the figure reflects the asymmetry of the keyhole in the longitudinal direction. In the spectrum of a secondary signal in this case, the first harmonic of the signal prevails. The lag (shift) of the secondary signal relative to the current of the deflection coils can be noted (Δ*τ*_0_ or Δ*S*).

**Figure 7.**
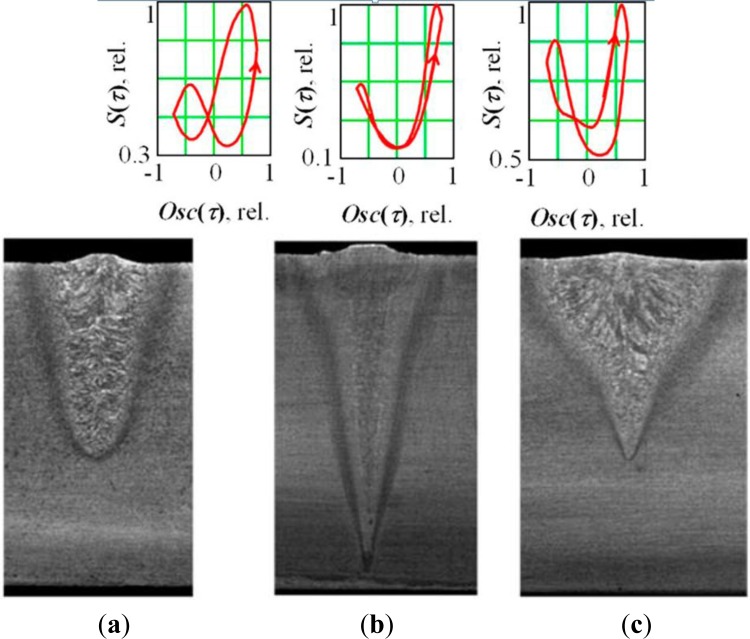
Results of coherent accumulation at the steel welding with oscillation along the joint for different focus regime. (**a**) underfocused (Δ*I_f_* = − 20 mA); (**b**) sharp focus (Δ*I_f_* = 0); (**c**) overfocused regimes (Δ*I_f_* = +15 mA) (P = 3 kW, oscillation frequency f = 645 Hz, sweep size 2A = 1.5 mm).

The signal shift Δ*τ*_0_ (Δ*S*) is more obvious in under-focused and over-focused modes and is zero in sharp focusing regime (Figure 7). If we compare their relationship, we see an increase of the second harmonica on transition to the over-focused modes. We see that under an under-focused regime, the beam interacts with a forward wall and the bottom of the keyhole; whereas, under an over-focused regime, it interacts with the forward and rear walls almost equally.

The lag value Δ*τ*_0_ monotonously decays once the focusing current rises, becoming positive upon insufficient focusing or negative upon excessive focusing. This allows identification of the electron-beam focusing mode at EBW without application of additional low-frequency scanning of the focus. This can be used to work out operational control methods for electron-beam focusing at the welding process. These results were found in the whole range of researched modes. To define the exact type of the dependency, we carried out a multi-factor experiment with a planning matrix of 27 experimental values. Regression equations were written as functions of factors from Table 1 for the parameter Δ*τ*_0_. The regression equation for the parameter Δ*τ*_0_ expressed in absolute units is:

(4) $$\begin{array}{l} \Delta \tau_{0}=-3.35\cdot 10^{-4}-2.94\cdot 10^{-5}\cdot \Delta I_{f}+8.52\cdot 10^{-5}\cdot \left(2A\right)+6.27\cdot 10^{-7}\cdot f+2.43\cdot 10^{-8}\cdot \Delta I_{f}\cdot f\\ -1.05\cdot 10^{-7}\cdot \left(2A\right)\cdot f-1.67\cdot 10^{-10}\cdot f^{2}\;\;\;\;\;\;\;\;\;\;\;\;\left(s\right) \end{array}$$

One should note that the coefficient of Δ*I_f_^2^* is statistically non-significant. Therefore, we can consider the dependency of Δ*τ*_0_ from the focusing mode Δ*I_f_* in the sharp focusing area as near to linear, assuming that all other factors are constant. The reliability of this model has been proved by mathematical analysis: the Fisher criterion was *F* = 28.9 and the hypothesis refusal probability was 10^−8^. The coefficient of correlation was 0.957, which demonstrates the presence of a direct relationship between the resulting function and the observed parameters. It should be noted that this dependence is applicable near the sharp focus regime only, when there is sufficiently intense evaporation from the interaction area of the electron beam with the metal.

**Figure 8.**
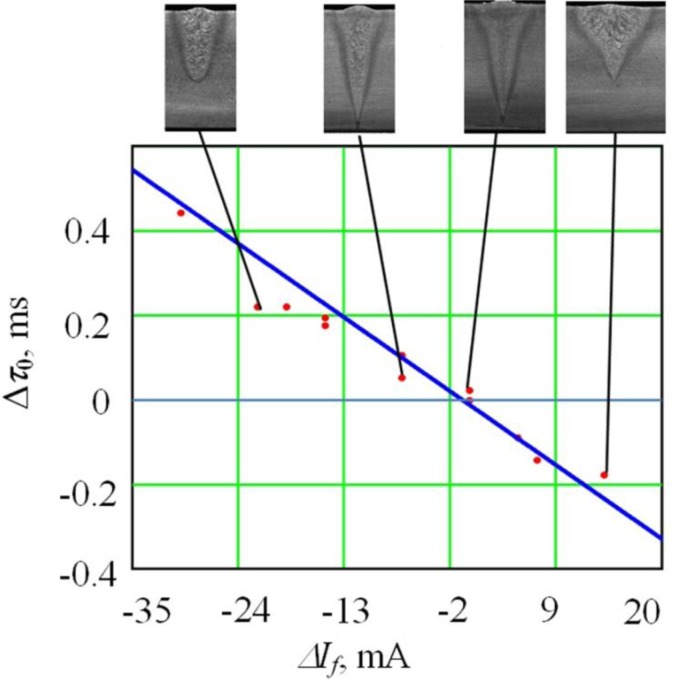
Connection of the shift value Δ*τ*_0_ (delay of function *S*(*τ*) concerning signal in deflection coils) with focus range Δ*I_f_*. Steel welding with the oscillation along the joint (power *P* = 3 kW, oscillation frequency *f* = 645 Hz, sweep size 2*A* = 1.5 mm).

To prove the created model, we carried out 12 additional experiments. In this case the focus degree was varied; the frequency and amplitude did not change. The results of the additional experiments along with values obtained from Equation (4) are shown in Figure 8. The error ratio was 0.066 with reliability equal to 0.95.

## 4. Conclusions

The results of this study demonstrate the possibility of understanding the processes in the penetration channel in EBWbased on the parameters of plasma secondary-emission electron current signal formed above the welded zone. It is possible to state the following:

1. A duplicated harmonica of the oscillation frequency dominates in plasma secondary current signal spectrum at EBW with electron-beam oscillation across the joint.
2. Both first and second harmonics are present at the process of EBW with electron-beam oscillation along the joint in the plasma secondary current signal. With increasing focus degree the first harmonic decreases monotonously and the second harmonic increases.
3. The method described here makes it possible to observe the shape of the keyhole directly in the welding regime. In the underfocused regime, the beam interacts with a forward wall and the bottom of the keyhole. In the overfocused regime, the keyhole becomes more symmetric and the beam interacts almost equally with the forward and back walls of the keyhole.
4. The results obtained by the coherent accumulation method function *S*(*τ*) have a lag (time shift) in relation to the signal of deflection coils. The lag value (Δ*τ*_0_) monotonously depends on focus regime and changes its sign when transferring from the underfocused to the overfocused condition. This fact allows identification of the electron-beam focus regime electron without additional low-frequency scanning of the focus. It can be used to work out operational control methods for electron-beam focusing at the welding process.

## Figures and Tables

**Table 1.** Factor variations in the experiment

**Series**	**Cross Sweep**				**Along Sweep**			
Factors	Δ*I_f_*, mA	*f*, Hz	2*A*, mm	*P*, kW	Δ*I_f_*,mA	*f*, Hz	2*A*, mm	*P*, kW
Lower limit of variation	−24	200	0.1	2	−24	90	0.4	2
Upper limit of variation	24	2000	1.4	4	24	1200	2.7	4
